# News from the Fungal Front: Wall Proteome Dynamics and Host–Pathogen Interplay

**DOI:** 10.1371/journal.ppat.1003050

**Published:** 2012-12-27

**Authors:** Clemens J. Heilmann, Alice G. Sorgo, Frans M. Klis

**Affiliations:** Swammerdam Institute for Life Sciences, University of Amsterdam, Amsterdam, The Netherlands; Duke University Medical Center, United States of America

## Introduction

In *Candida albicans*, like in *Saccharomyces cerevisiae*, the basal layer of the mature cell wall consists of a network of β-1,3- and β-1,6-glucans and chitin and functions as a skeletal layer. This basal layer is covered by an external layer of highly glycosylated, covalently anchored wall proteins radiating from the cell surface, which are directly involved in the first contacts between the fungal pathogen and host cells. The majority of the covalently bound wall proteins are modular glycosylphosphatidylinositol (GPI)-proteins. In their final form, wall-bound GPI-proteins usually consist of a C-terminal, truncated GPI-anchor that attaches them to the β-glucan layer, followed by a heavily glycosylated serine/threonine-rich spacer domain that often includes repeats, and an N-terminally located functional domain protruding from the cell surface [1]. At any given time-point >20 different covalently bound wall proteins can be identified [2], [3] that are involved in processes such as adhesion, biofilm formation, wall remodeling, iron acquisition, and coping with immune responses. Importantly, the wall proteome is highly dynamic and continuously adapts to the specific conditions that *C. albicans* encounters in the host environment. In this review we examine the role of wall proteins in infection-related processes and assess their potential as targets for antifungal and vaccine development.

## Why Do Most Wall Proteins Form Families?

**Figure 1:**
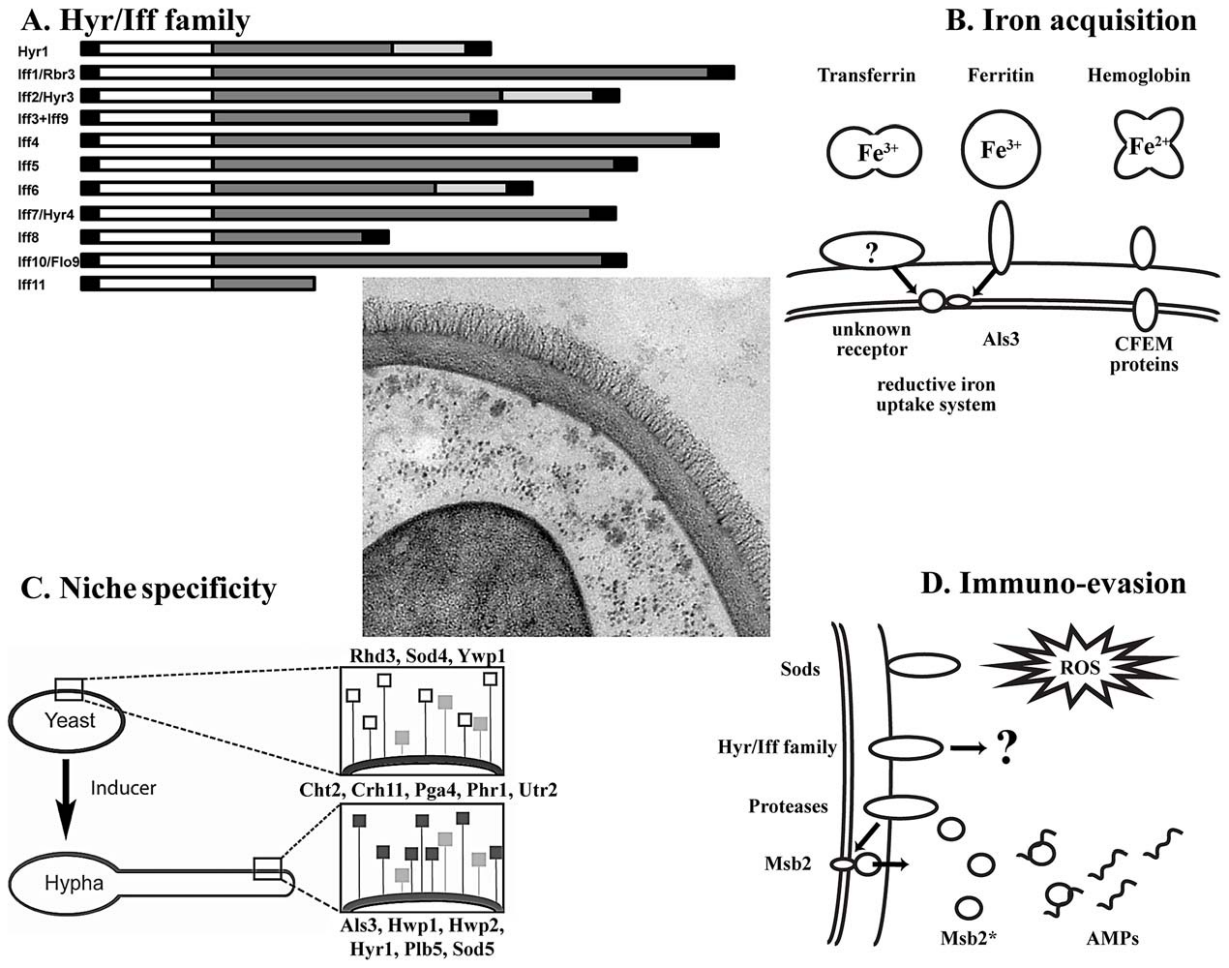
Key concepts of the *C. albicans* wall proteome. Center: TEM picture of the cell wall and its proteins (courtesy of Iuliana V. Ene and Alistair J.P. Brown, Aberdeen). (A) Domain structure of the Hyr/Iff family (adapted from [7]). From left to right: N-terminal signal peptide; white box, conserved domain; dark grey box, Ser/Thr-rich region; light grey box, Asp/Gly-rich region; black box, GPI-anchor addition signal. (B) Wall proteins implicated in iron acquisition from host proteins. Membrane and wall-bound CFEM proteins are able to bind hemoglobin, while Als3 is the receptor for ferritin. It is unknown if there exists a receptor for transferrin. Bound hemoglobin is taken up by endocytosis, while iron from ferritin and transferrin is sequestered via the reductive iron uptake system. (C) Effect of yeast-to-hypha transition on the wall proteome with yeast-associated (top; open squares), morphotype-independent (middle; grey squares) and hypha-associated (bottom; black squares) proteins [2]. (D) Interaction of wall proteins with the immune system. Wall-resident superoxide dismutases (Sods) detoxify reactive oxygen species (ROS) to H_2_O_2_, which is subsequently converted into H_2_O and O_2_ by catalase activity [20]. Proteins of the Hyr/Iff family confer resistance to neutrophil and phagocyte killing through an unknown mechanism [12]. Possibly, like in *S. cerevisiae*, proteases situated on the cell wall process the trans-membrane signaling protein Msb2 and liberate the extracellular domain Msb2*. Msb2* is able to bind to antimicrobial peptides (AMPs) in a dose-dependent manner and confers resistance [21].

*C. albicans* is able to thrive in many host niches, including the skin, mucosal surfaces, the bloodstream, and internal organs. Wall proteins are subject to the surrounding conditions and come into contact with highly diverse, niche-associated, extracellular matrix proteins from the host as well as with bacterial surface proteins. This probably explains the evolution of many wall protein families with individual members showing optimal functionality dependent on environmental conditions and infection sites [1]. For example, the environmental pH strongly affects the wall proteome, revealing the preferred usage of specific family members at acidic and neutral pH [4]. Interestingly, invasive growth is generally associated with hyphal growth, and comparison of the wall proteomes of yeast and hyphal cells revealed a core set of hypha-associated wall proteins under various hyphal growth-inducing conditions (Als3, Hwp1, Hwp2, Hyr1, Plb5, and Sod5) [2], [5]. The two largest wall protein families are the Als family [6] and the Hyr/Iff family [7]. The family of agglutinin-like sequence (ALS) proteins consists of eight GPI-modified, elongated, broad-specificity adhesins with an immunoglobulin-like N-terminal domain that can interact with a wide variety of host proteins [8]. Some Als proteins possess amyloid-forming sequences, which could play a role in forming biofilms [9]. Fascinatingly, Als3 has multiple functions, including ferritin binding [10] as well as binding to E-cadherin, thereby facilitating iron uptake and active internalization of *C. albicans* by host cells, respectively [11]. This supports that proteins of a family may share a particular function, but might also have additional functions that are not conserved throughout the family. Intriguingly, Hyr1, one of the 12 GPI-proteins belonging to the Iff/Hyr family, is strongly hypha-associated and confers resistance to neutrophil killing [12] through its N-terminal domain. Although the domain structure within the family is variable, the N-terminal domain is strongly conserved in all family members (Figure 1) [7]. This hints at a more general, niche-specific role of the family in evading immune cells under different growth conditions.

## What Is the Role of Wall Proteins in Iron Acquisition?

One of the most restricted nutrients in the human body is iron. Because of its reactive nature, but also in order to restrict growth of invading microorganisms, free iron is highly limited in the host and mainly found in association with proteins, either as a prosthetic group like in hemoglobin and myoglobin, stored inside ferritin, transported by transferrin, or liganded by lactoferrin. *C. albicans* has evolved a number of strategies to scavenge iron from these complexes. Of the five Rbt5 family proteins, which belong to the CFEM superfamily and are characterized by an internal domain containing eight invariantly spaced cysteines [13], Csa1, Pga7, Pga10, and Rbt5 are found attached both to the plasma membrane and the wall, while Csa2 is secreted [3], [14]–[16]. It has been shown that Csa1, Pga10, and Rbt5 are involved in heme binding [17]. As the expression of *CSA1*, *CSA2*, *PGA7*, *PGA10*, and *RBT5* is co-regulated under various conditions, including iron restriction, the question arises whether the Rbt5 family proteins might act as a relay system, similar to bacterial iron uptake systems [18]. As mentioned above, Als3 is also important for iron acquisition as a receptor for ferritin, an iron-storage host molecule that contains about 30% of the total human iron pool. Without Als3, *C. albicans* is unable to grow with ferritin as its sole iron source [10].

## Which Wall Proteins Allow *C. albicans* to Cope with the Host Immune Response?

*C. albicans* has evolved various mechanisms to avoid or counteract the immune response. The cell wall is the first line of defense, but also a target for the immune system due to its immunogenic epitopes. For example, the receptor dectin-1, which is mainly expressed on dendritic cells and macrophages, recognizes the β-glucan of the wall and leads to the activation of pro-inflammatory cytokines [19]. However, the mannoprotein coat largely prevents the detection of the underlying β-glucan layer. Additionally, the wall protein Hyr1 effectively reduces immune cell killing of *C. albicans* [12]. In support of its protective role, heterologous expression of Hyr1 in *Candida glabrata* also mitigates immune cell killing, suggesting a direct function of the protein. *C. albicans* also has two wall-bound, morphotype-associated superoxide dismutases (Sod4, Sod5) [14]. These cell wall–resident superoxide dismutases (Sods) are essential for dealing with extracellular ROS (reactive oxygen species), resulting from the oxidative burst, a general mechanism of immune cells to kill invading pathogens. As expected, *SOD4* and *SOD5* knockout mutants are more susceptible to oxidative stress [20]. Sod6, another GPI-anchored member of the Sod family, has not been detected in proteomic screens, and gene deletion did not reveal a clear phenotype [20]. Antimicrobial peptides, like histatins, defensins, and cathelicidins, belong to the arsenal of host defense mechanisms as well. Recently, the shedding of the extracellular part of the plasma membrane-bound signaling mucin Msb2, which is involved in maintaining cell wall integrity, has been shown to convey resistance to histatin-5 and the cathelicidin LL-37 in a dose-dependent manner [21]. This processing and shedding is likely mediated by secretory aspartyl proteases (Saps) [22], but there is no evidence that the GPI-modified, wall-resident proteins Sap9 and Sap10 are involved [21], [22].

## How Do the Wall and its Proteins Cope with Surface Stress?

Cell shape is mainly determined by the skeletal polysaccharides of the wall, which are important for resisting the internal turgor pressure and shielding the cell from external mechanical forces. Nonetheless, remodeling of the wall is required, for example, during isotropic growth and cell separation, and for coping with surface stress. Remodeling of the wall is mediated by wall- and plasma membrane-resident, carbohydrate-active enzymes that detach, re-arrange, and re-attach carbohydrates. The main wall-bound proteins involved are a chitinase (Cht2), transglucosylases (Phr1, Phr2, Pga4), and chitin transglycosylases (Crh11, Utr2). The secretory aspartyl proteases Sap9 and 10, and Pir1, a predicted β-glucan cross-linking protein, also seem to be involved [3], [23]. In contrast to Sap1 to 8, Sap9 and 10 are GPI-modified, yapsin-like proteases that are retained at the cell surface [23]. Interestingly, Sap9 has been implicated in the processing and shedding of other wall proteins, most notably, the chitinase Cht2 and Pir1 [24]. The levels of wall-bound Sap9 seem largely morphotype-independent, but its levels increase in conjunction with surface stress conditions as observed in response to fluconazole [3].

Strikingly, when *C. albicans* is grown on a poor carbon source such as lactate (found in the vaginal fluid and together with acetate maintaining its acidic pH [4]), or on a mixture of lactate and glucose, the cell wall gets significantly thinner and more flexible. Importantly, these alterations are accompanied by substantial changes in the wall proteome [25]. This and other studies have identified a core set (Crh11, Phr1, Phr2, Pga4, Sap9, Utr2) of wall-remodeling proteins that is conserved in the response to several surface-stress conditions ([3], [25] and (Heilmann et al., unpublished data). Conceivably, this protein set could also be important to survive other surface stresses, including membrane-perturbing antimicrobial peptides found in body fluids, epithelial layers, and immune cells. The functional domains of these proteins are conserved in the Ascomycotina, suggesting similar importance for other fungi as well.

## Which Wall Proteins Are Promising Targets for Vaccine Development?

A vaccine that could be administered to high-risk groups, e.g., pre-surgery, or to women suffering from recurrent vaginitis, would be an important asset. As stated earlier, the functional domain of wall proteins is almost exclusively situated in the N-terminal region, while the C-terminal part is mainly of structural importance. This is reflected in the various vaccines that are currently being developed (reviewed in [1], [26]). For example, mice immunized with the recombinantly expressed N-terminal domain of Als3 become resistant to infections by *C. albicans* as well as *Staphylococcus aureus* [27]. The N-terminal domains of Als1 and Hyr1, and a short immunogenic peptide from the N-terminal domain of Hwp1 conjugated to a β-1,2-linked mannotrioside, have been used similarly as *C. albicans* vaccines [1], [12]. Notably, these four vaccine targets are strongly associated with hyphae, suggesting that hyphal epitopes might be more easily recognized by the immune system as a threat, since they are associated with the breaching of host tissue. Invasive growth in vivo is not only associated with hyphal growth, but probably also with iron restriction and thus with increased levels of the iron acquisition proteins in the wall [15]. Relevantly, all five members of the Rbt5 family contain an identical sequence (with Csa1 containing four copies) that could represent a prime target. Developing this approach further, it is conceivable to combine immunogenic epitopes from the N-terminal functional region of a selection of wall proteins in a single recombinant protein for use as a multi-component vaccine. In summary, the evolution of wall protein families in the human fungal pathogen *C. albicans* allows survival in diverse host niches and has resulted in an impressive plasticity of the wall proteome. The exposure of wall proteins on the surface together with their critical functions, and the use of single- or multi-component vaccines, makes them promising targets for combating fungal infections.
